# Chromosome 9p21 and ABCA1 Genetic Variants and Their Interactions on Coronary Heart Disease and Ischemic Stroke in a Chinese Han Population

**DOI:** 10.3390/ijms17040586

**Published:** 2016-04-18

**Authors:** Xiao-Li Cao, Rui-Xing Yin, Feng Huang, Jin-Zhen Wu, Wu-Xian Chen

**Affiliations:** 1 Department of Cardiology, Institute of Cardiovascular Diseases, the First Affiliated Hospital, Guangxi Medical University, 22 Shuangyong Road, Nanning 530021, Guangxi, China; maten1996@gmail.com (X.-L.C.); huangfeng3000@126.com (F.H.); wujinzhengx@sohu.com (J.-Z.W.); nncwx@163.com (W.-X.C.); 2 Department of Neurology, the First Affiliated Hospital, Guangxi Medical University, 22 Shuangyong Road, Nanning 530021, Guangxi, China

**Keywords:** coronary heart disease, ischemic stroke, chromosome 9p21, adenosine triphosphate (ATP)-binding cassette transporter A1, single nucleotide polymorphism

## Abstract

The single nucleotide polymorphisms (SNPs) related to both coronary heart disease (CHD) and ischemic stroke (IS) in Chinese individuals have not been identified definitely. This study was developed to evaluate the genetic susceptibility to CHD and IS on the chromosome 9p21 and the adenosine triphosphate (ATP)-binding cassette transporter A1 genes (*ABCA1*) in a Chinese Han population. Genotypes of the rs1333040, rs1333042, rs4977574, rs2066715 and rs2740483 SNPs were determined in 1134 unrelated patients (CHD, 565 and IS, 569) and 541 controls. The frequencies of the rs4977574 genotypes and alleles between CHD and control groups, and the rs2740483 genotypes and alleles between IS and control groups were different (*p* = 0.006–0.001). The subjects with rs1333042GG genotype and the carriers of the rs4977574G allele were associated with increased risk of CHD. The carriers of the rs4977574G allele were associated with increased risk of IS. However, the carriers of the rs2740483C allele had lower risk of IS than the non-carriers of the rs2740483C allele after controlling for potential confounders. The rs4977574GG-age (>60 year) interaction increased the risk of CHD (*p* = 0.022), whereas the rs2740483CG/CC-body mass index (>24 kg/m^2^) interaction decreased the risk of IS (*p* = 0.035). The interactions of rs1333040-rs1333042 on the risk of CHD and IS were relatively strong, whereas the interactions of rs1333040-rs1333042-rs2066715 and rs1333040-rs1333042-rs2066715-rs2740483 on the risk of CHD, and rs1333040-rs1333042-rs4977574 and rs1333040-rs1333042-rs4977574-rs2740483 on the risk of IS were relatively weak. These findings suggest that some common variants on the chromosome 9p21 and *ABCA1* and their interactions may significantly modify the risk of CHD and IS independent of effects on serum lipid levels.

## 1. Introduction

Coronary heart disease (CHD) and ischemic stroke (IS) remain the leading causes of morbidity and mortality in both developed and developing countries at present [1,2]. More than 2.5 and 1 million persons are affected by stroke and heart attack, respectively, leading to more than 2 million deaths each year in China [3]. Both CHD and IS are multifactorial diseases in which multiple genes and environmental factors are involved. Atherosclerosis, a progressive inflammatory disorder, has been found to be the pathological basis of both diseases. Therefore, CHD and IS may share some common genetic and environmental determinants such as sex, age, dyslipidemia, hypertension, diabetes, smoking behavior, and family history [2,3,4]. Recent genome-wide association studies (GWASes) have identified several genes and loci in the predisposition to CHD [5] or IS [6] in different populations. Furthermore, some single nucleotide polymorphisms (SNPs) originally identified as influencing the risk of CHD were also subsequently associated with IS [7,8]. Heritability estimates of coronary artery calcification are approximately 50% [9,10]. In fact, twin and family studies have indicated that the heritable factors account for 30%–60% of the interindividual variation in the risk of CHD [11,12]. A recent GWAS conducted by Bevan *et al*. [13] illustrated that heritability was 37.9% for all IS, 40.3% for large-vessel disease, 32.6% for cardio-embolic, and 16.1% for small-vessel disease. Although data accumulated from previous studies have identified associations between SNPs on chromosome 9p21 and the risk of CHD [14,15,16,17,18] and IS [19,20] in Caucasians, little is known about such associations among the Chinese populations. Moreover, many SNPs have still not been detected on the chromosome 9p21 region.

The adenosine triphosphate (ATP)-binding cassette (ABC) transporter A1 (ABCA1) is a member of the ABC family of proteins that are involved in the transmembrane transport of a wide variety of substrates [21]. ABCA1 can promote efflux of cellular cholesterol from macrophages and other peripheral tissues to apolipoprotein (Apo) acceptors. The ABCA1 gene (*ABCA1*) is located on chromosome 9q31.1. It contains 49 exons that range in size from 33 to 249 bp [22]. Several common SNPs in this gene have been associated with susceptibility to CHD [23,24] and IS [25] in the general population, but several studies have reported apparently conflicting results [26,27]. In this study, we investigated whether three SNPs of rs1333040, rs1333042 and rs4977574 on chromosome 9p21 and two common SNPs of rs2066715 and rs2740483 in the *ABCA1* and their interactions have an association with the risk of CHD and IS in a Chinese Han population.

## 2. Results

### 2.1. Clinical Characteristics

The clinical characteristics of the subjects are presented in Table 1. The values of body mass index (BMI) and serum triglyceride (TG), and the prevalence of diabetes and hypertension were higher but the levels of serum total cholesterol (TC), high-density lipoprotein cholesterol (HDL-C), ApoA1, and the ratio of ApoA1 to ApoB were lower in both CHD and IS patients than in controls (*p* < 0.05–0.001). The prevalence of hyperlipidemia was higher in CHD than in controls (*p* < 0.05), whereas it was lower in IS patients than in controls (*p* < 0.001).

### 2.2. Polymerase Chain Reaction (PCR) Products and Genotypes

**Figure 1:**
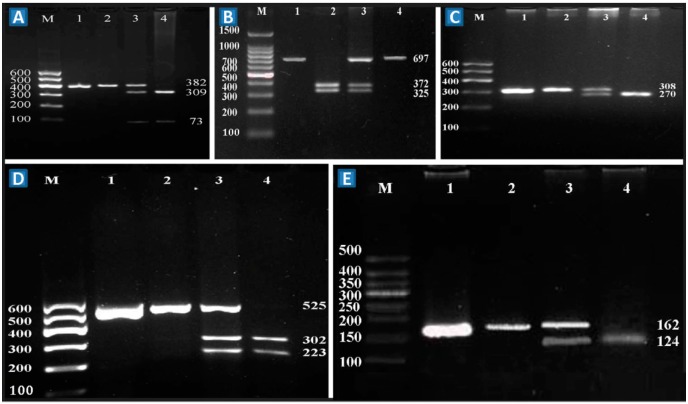
Genotyping of the rs1333040, rs1333042, rs4977574, rs2066715 and rs2740483 SNPs on the chromosome 9p21 and *ABCA1*. **A**–**E** represent the rs1333040, rs1333042, rs4977574, rs2066715 and rs2740483 SNPs; respectively. Lane M, 100 bp marker ladder; lane 1, the PCR products; lines A2, B2, C2, D2 and E2 were rs1333040TT (382 bp), rs1333042GG (325- and 372-bp), rs4977574AA (308 bp), rs2066715GG (525 bp), and rs2740483GG (162 bp) genotypes, respectively; lines A3, B3, C3, D3 and E3 were rs1333040CT (73, 309 and 382 bp), rs1333042AG (325, 372 and 697 bp), rs4977574AG (270 and 308 bp), rs2066715AG (223, 302 and 525 bp), and rs2740483CG (124 and 162 bp) genotypes, respectively; lines A4, B4, C4, D4 and E4 were rs1333040CC (73 and 309 bp), rs1333042AA (697 bp), rs4977574GG (270 bp), rs2066715AA (223 and 302 bp), and rs2740483CC (124 bp) genotypes, respectively.

The polymerase chain reaction (PCR) products of the rs1333040, rs1333042, rs4977574, rs2066715 and rs2740483 SNPs were 382-, 697-, 308-, 525- and 162-bp nucleotide sequences, respectively (Figure 1, lane 1). The genotypes identified were named according to the absence or presence of the enzyme restriction sites (Figure 1, lanes 2 to 4).

### 2.3. Nucleotide Sequences

**Figure 2:**
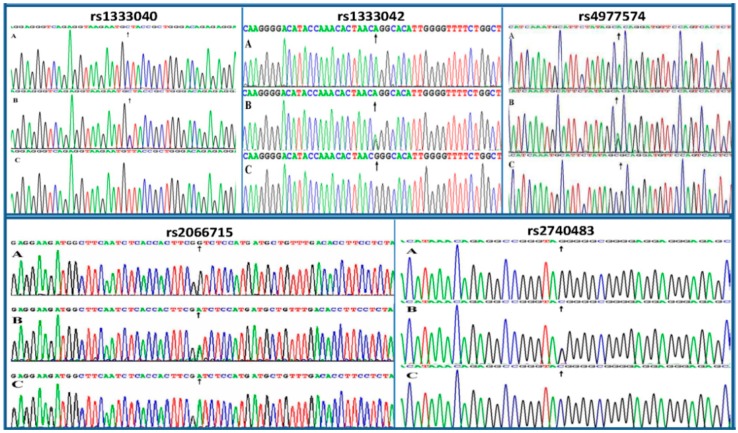
A part of the nucleotide sequences of the rs1333040, rs1333042, rs4977574, rs2066715 and rs2740483 SNPs on the chromosome 9p21 and *ABCA1*. **A** was rs1333040CC, rs1333042AA, rs4977574AA, rs2066715GG, and rs2740483GG genotypes, respectively; **B** was rs1333040CT, rs1333042AG, rs4977574AG, rs2066715AG, and rs2740483CG genotypes, respectively; **C** was rs1333040TT, rs1333042GG, rs4977574GG, rs2066715AA, and rs2740483CC genotypes, respectively. Black arrows, the variation nucleotides.

The genotypes of the rs1333040, rs1333042, rs4977574, rs2066715 and rs2740483 SNPs detected by the PCR-RFLP were also confirmed by direct sequencing (Figure 2), respectively.

### 2.4. Genotypic and Allelic Frequencies

The frequencies of the rs1333040, rs1333042, rs4977574, rs2066715 and rs2740483 genotypes and alleles are summarized in Table 2. The genotype distribution of the five SNPs was consistent with the Hardy-Weinberg equilibrium in patients and controls (*p*_HWE_ > 0.05 for all). The frequencies of the rs4977574 genotypes and alleles between CHD patients and controls, the frequencies of the rs2740483 genotypes and alleles, and the frequencies of the rs4977574 alleles between IS patients and controls were different (*p =* 0.006–0.001). There was no difference in the frequencies of the rs1333040, rs1333042 and rs2066715 genotypes and alleles between CHD or IS patients and controls (*p* > 0.01 for all).

### 2.5. Genotypes and the Risk of Coronary Heart Disease (CHD) and Ischemic Stroke (IS)

As shown in Table 3, five genetic models were used to detect the genetic associations of the SNPs and the risk of CHD and IS. The SNPs of rs1333042 and rs4977574 were associated with increased risk of CHD after controlling for potential confounders including age, sex, BMI, cigarette smoking, hypertension, diabetes and hyperlipidemia. The subjects with rs1333042GG (Codominant: OR = 1.92, 95% CI = 1.20–3.08, *p* = 0.007) and rs4977574GG (Codominant: OR = 1.64, 95% CI = 1.14–2.37, *p* = 0.008) or rs4977574AG/GG (Dominant: OR = 1.52, 95% CI = 1.11–2.07, *p* = 0.009) genotypes had higher risk of CHD than those with the rs1333042AA and rs4977574AA genotypes, respectively.

The rs4977574 and rs2740483 genotypes were associated with the risk of IS after controlling for potential confounders. The subjects with the rs4977574GG (Codominant: OR = 1.63, 95% CI = 1.14–2.33, *p* = 0.007) or rs4977574AG/GG (Dominant: OR = 1.51, 95% CI = 1.12–2.03, *p* = 0.007) genotypes had higher risk of IS than those with the rs4977574AA genotypes, respectively. However, the carriers of the rs2740483C allele had lower risk of IS than the non-carriers of the rs2740483C allele after controlling for potential confounders (Codominant: GG *vs*. CG, OR = 0.64, 95% CI = 0.48–0.85, *p* = 0.002; GG *vs*. CC, OR = 0.22, 95% CI = 0.10–0.49, *p* < 0.001; Dominant: OR = 0.58, 95% CI = 0.44–0.76, *p* < 0.001).

### 2.6. Linkage Disequilibrium (LD) Analyses

The LD in CHD patients was weak among the rs1333040, rs1333042 and rs4977574 SNPs: *D′* = 0.406 for rs1333040 and rs1333042, *D′* = 0.364 for rs1333040 and rs4977574, and *D′* = 0.552 for rs1333042 and rs4977574. The LD in IS patients was also weak among the rs1333040, rs1333042 and rs4977574 SNPs: *D′* = 0.616 for rs1333040 and rs1333042, *D′* = 0.504 for rs1333040 and rs4977574, and *D′* = 0.455 for rs1333042 and rs4977574. Thus, haplotype analyses among the SNPs and the associations between the haplotypes and the risk of CHD and IS were not performed (*D′* < 0.7) in this study.

### 2.7. Gene-Environment Interactions on the Risk of CHD and IS

The interactions of gene-environment on the risk of CHD and IS are shown in Table 4. The genotype of rs4977574GG when interacted with age (>60 year) increased the risk of CHD (*p*_CHD_ = 0.022), whereas the genotypes of rs2740483CG/CC when interacted with BMI (>24 kg/m^2^) decreased the risk of IS (*p*_I__S_ = 0.035).

### 2.8. Gene–Gene Interactions on the Risk of CHD and IS

**Figure 3:**
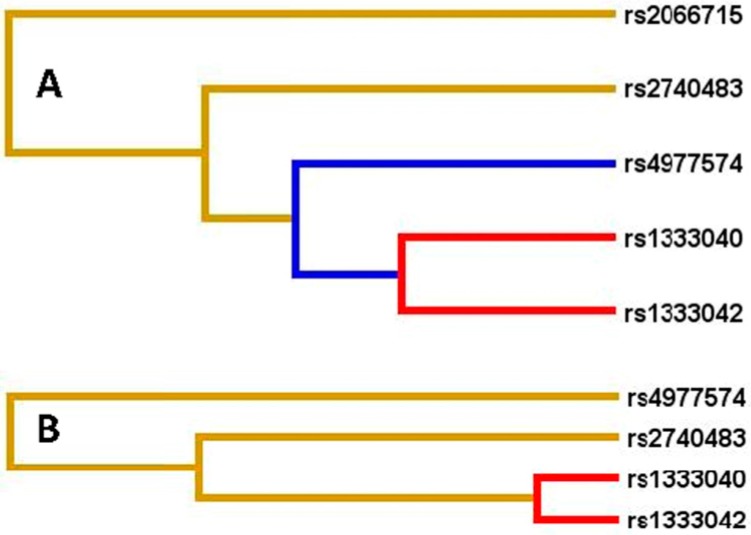
Diagram for gene–gene interactions on the risk of CHD (**A**) and IS (**B**). Red color, strong interaction; blue color, moderate interaction; yellow color, weak interaction.

The interactions of gene–gene on the risk of CHD and IS are displayed in Figure 3 and Table 5 and Table 6. The interactions of the rs1333040-rs1333042 on the risk of CHD and IS were relatively strong, whereas the interactions of the rs1333040-rs1333042 and rs2066715 (rs1333040-rs1333042-rs2066715) and rs2740483 (rs1333040-rs1333042-rs2066715-rs2740483) on the risk of CHD, and the rs1333040-rs1333042 and rs4977574 (rs1333040-rs1333042-rs4977574) and rs2740483 (rs1333040-rs1333042-rs4977574-rs2740483) on the risk of IS were relatively weak (interaction strength: red color > blue color > yellow color).

## 3. Discussion

The principal findings of the present study are that several SNPs on the chromosome 9p21 and *ABCA1* and their interactions associate strongly with the risk of CHD and IS in a Chinese Han population. The frequencies of the rs4977574 genotypes and alleles on chromosome 9p21 were different between CHD patients and controls (*p =* 0.006–0.001). Genetic association analyses also showed that the SNPs of rs1333042 and rs4977574 were associated with increased risk of CHD after controlling for potential confounders including age, sex, BMI, cigarette smoking, hypertension, diabetes and hyperlipidemia. The subjects with the rs1333042GG and rs4977574GG or AG/GG genotypes had higher risk of CHD than those with the rs1333042AA and rs4977574AA genotypes.

Several previous studies have shown that the SNP of rs1333040 was significantly associated with the risk of CHD in North Indian [28], German [18], and Chinese Taiwanese [29] populations, but not in Iran [30]. In the current study, we failed to show any association of the rs1333040 SNP and the risk of CHD. The reason for these contradictory results is unclear. Significant differences in the genetic variation and in the LD patterns among populations probably explain the population disparities in the susceptibility to CHD. For example, three CHD-related SNPs of rs9632884, rs1537371 and rs1333042 on the chromosome 9p21 region showed consistent signals of positive selection in populations from Europe, but not from the Middle East, North Africa, Asia and Africa [31]. Therefore, natural selective processes may be the cause of some of the population differences detected for specific genetic variants.

The SNP of rs4977574 is a non-protein-coding SNP (A > G). Several GWASes and replication studies have shown a consistent association of this SNP and the risk of CHD in populations of European or Eastern Asian descent [32,33,34,35]. In the present study, our findings are consistent with those of previous studies [32,33,34,35].

Tan *et al*. [36] showed that the SNP of *ABCA1* rs2066715 was clearly associated with CHD status in Malays but not in Indians and Singapore Chinese; the SNP could predict increased risk of CHD [23]. Functional analyses showed that the rs2066715 (V825I) SNP influenced the *ABCA1* function and that the 825I variant had higher activity in mediating cholesterol efflux than the wild 825V. The carriers of 825I allele tended to have higher symptom onset age. These data indicate that there may be an effect of common *ABCA1* functional variants on age of symptom onset in the patients with CHD [37].

In a sample of 171 men who survived myocardial infarction before 45 years, no difference in the *ABCA1* rs2740483 (C-17G) allele frequencies was found between the patients and controls [38]. Zwarts *et al*. [39] showed that the SNP of rs2740483 was associated with decreased coronary events without necessarily influencing blood lipid levels. Regieli *et al*. [40] prospectively investigated the effects of *ABCA1* SNPs on long-term clinical outcome in CHD patients. The results demonstrated that the protection from 10-year vascular death could be attributed to the SNP of rs2422493 and the haplotype of rs2422493T-rs1800976G-rs2740483C-rs1800977C (hazard ratio = 0.53, *p* = 0.04). The haplotypes of rs2422493T-rs1800976G-rs2740483C-rs1800977C (*p* = 0.04) and rs2422493T-rs1800976C-rs2740483C-rs1800977T (*p* = 0.003) exhibited less extensive CHD.

In this study, we also showed that the frequencies of the rs4977574 alleles on chromosome 9p21, and the frequencies of the *ABCA1* rs2740483 genotypes and alleles, were different between IS patients and controls (*p =* 0.004–0.001). The SNP of rs4977574 was associated with increased risk of IS, whereas the SNP of rs2740483 was associated with decreased risk of IS after controlling for potential confounders. The carriers of the rs4977574G allele had higher risk of IS than the non-carriers of the rs4977574G allele (*p* = 0.001), whereas the carriers of the rs2740483C allele had lower risk of IS than the non-carriers of the rs2740483C allele (*p* < 0.001). To the best of our knowledge, this is the first report on the association of the rs2740483 SNP and IS.

In a recent study, Heckman *et al*. [41] found that four SNPs on chromosome 9p21 were significantly associated with the risk of cardio-embolic stroke in Caucasians, the strongest of which was rs1333040 SNP (OR = 1.55, *p* = 0.0007). They were also associated weakly with small vessel stroke. There were no associations between the observed SNPs and large vessel stroke. The SNPs of rs1333040 and rs1333042 on chromosome 9p21 were also associated with the risk of IS in African Americans (OR = 0.65, *p* = 0.023 and OR = 0.55, *p* = 0.070, respectively).

Lövkvist *et al*. [42] showed that the SNP of rs4977574 on chromosome 9p21 was associated with overall IS (OR = 1.12; 95% CI: 1.04–1.20; *p* = 0.002) as well as large vessel stroke (OR = 1.36; 95% CI: 1.13–1.64; *p* = 0.001). Recently, Lu *et al*. [43] found that genetic variation of rs4977574 was potentially associated with noncardioembolic cerebral infarction and carotid plaque in the Chinese Han population. The frequency of the rs4977574G allele was higher in patients with cerebral infarction without carotid plaque than in control middle-aged patients (OR = 1.519, *p* = 0.013). The risk of cerebral infarction was higher among the subjects with GG genotype than among the subjects with AG/AA genotypes (OR = 1.866, 95% CI = 1.088–3.201, *p* = 0.023). Among the patients with cerebral infarction aged >65 years, the SNP of rs4977574 was also associated with the risk of carotid plaque (OR = 1.997, *p* = 0.049). In a recent meta-analysis, however, Cheng *et al*. [44] showed that there was no association between the rs4977574 SNP and overall IS and/or stroke subtypes after adjusting for multiple comparisons.

The interactions of SNP-environment or SNP-SNP on chromosome 9p21 and *ABCA1* on the risk of CHD and IS have not been detected extensively. In the present study, we revealed that when the rs4977574GG genotype interacted with age (>60 year), it increased the risk of CHD, whereas the rs2740483CG/CC genotypes interacting with BMI (>24 kg/m^2^) decreased the risk of IS. The interactions of the rs1333040-rs1333042 on the risk of CHD and IS were relatively strong, whereas the interactions of the rs1333040-rs1333042-rs2066715 and rs1333040-rs1333042-rs2066715-rs2740483 on the risk of CHD, and the rs1333040-rs1333042-rs4977574 and rs1333040-rs1333042-rs4977574-rs2740483 on the risk of IS were relatively weak. This is the first report to demonstrate an interaction of the chromosome 9p21 and *ABCA1* SNP-environment or SNP-SNP on the risk of CHD and IS in a Chinese Han population.

Do *et al*. [45] have tested the interaction of the rs10757274, rs2383206, rs10757278 and rs1333049 SNPs on chromosome 9p21 and dietary intake on the risk of cardiovascular disease. There was a significant interaction between the rs2383206 SNP and factor-analysis-derived “prudent” diet pattern score (*p* = 4.0 × 10^−4^). These results suggest that the risk of CHD conferred by chromosome 9p21 SNPs was modified by a prudent diet high in raw vegetables and fruits. Hamrefors *et al*. [46] also observed an interaction between the rs4977574 SNP and smoking on incident CHD (*p* = 0.035) and cardiovascular disease mortality (*p* = 0.012). An interaction of the rs4977574 SNP and low omega-3 index on increased risk of acute coronary syndrome was also noted (OR = 1.57, 95% CI 1.07–2.32, *p* = 0.02), but this was not significant after correction for multiple testing [47].

Several potential limitations should be acknowledged in the present study. Firstly, the sample size is small and from a single hospital, and any interpretation should be done with caution. Secondly, there were significant differences in some clinical characteristics between the patients and controls. Although several confounders have been adjusted for the statistical analyses, we could not completely eliminate the potential influences of these factors on the results. Thirdly, the association of the rs1333040, rs1333042, rs4977574, rs2066715 and rs2740483 SNPs and serum lipid levels in CHD and IS patients was not analyzed because of the interference of lipid-lowering drugs. In a previous study, however, we showed that the rs2066715AA genotype was associated with higher TC levels in the Bai Ku Yao population (an isolated subgroup of the Yao minority in China), and lower HDL-C and ApoA1 levels in male Han Chinese than the GG and AG genotypes (*p* < 0.05 for all) [48]. Finally, it is now generally accepted that both CHD and IS are the complex disorders caused by multiple environmental and genetic factors and their interactions. Although we have detected the association of the rs1333040, rs1333042, rs4977574, rs2066715 and rs2740483 SNPs on chromosome 9p21 and *ABCA1* and the risk of CHD and IS, other genetic variants are not detected and analyzed together, and this may result in some misinterpretation of our results.

## 4. Materials and Methods

### 4.1. Study Population

This study included 1134 unrelated and consecutive patients with CHD (*n* = 565) and IS (*n* = 569). They were enrolled from September, 2009 to October, 2011. All of them were the inpatients from the First Affiliated Hospital, Guangxi Medical University. The age of the patients ranged from 31 to 92 years, with an average age of 62.31 ± 10.53 years in CHD and 62.53 ± 11.92 years in IS patients. The diagnosis of CHD was based on typical ischemic discomfort, electrocardiographic change, increases in the cardiac markers including creatinine kinase-MB and troponin T, as well as the outcomes of coronary angiography (coronary diameter ≥ 2 mm, stenosis ≥ 50%). Angiographic severity of disease was classified according to the number of coronary vessels with significant stenosis (luminal narrowing ≥ 50%) as one-, two-, or three-vessel disease in the three major coronary arteries [49,50]. The coronary angiograms were observed by two independent angiographers who were both blinded to the data of the genotypes. In the event of discordance of the number of vessels scored between the two angiographers, angiograms were scored by a third independent reviewer. The diagnosis and classification of IS were ascertained in accordance with the Trial of Org 10172 in Acute Stroke Treatment (TOAST) criteria [51] after strict clinical, laboratory and image examinations such as magnetic resonance imaging (MRI). The selected IS patients included large- and small-vessel stroke. Individuals with a history of cerebral hemorrhage, cardio-embolic stroke, neoplastic or intracranial space-occupying lesion, infection, and other types of intracranial lesions were excluded. The IS patients who had a past history of CHD, or the CHD cases who had a past history of IS were excluded from the study. There were 58 patients not included in this study because of the co-existence of both CHD and IS. Although CHD and IS have many common characteristics, they are two different diseases after all, and there are also many different aspects between the two diseases. They are not enough to become a subgroup for further analyses because the cases are too small.

A control group of 541 subjects matched by age, gender, and nationality was also included in this study. They were randomly selected from healthy adults who underwent periodical medical check-ups at our hospital during the same period when CHD and IS patients were recruited. The mean age of the controls was 61.39 ± 11.41 years. They were free of CHD and IS at time of history taking, clinical, biochemical, and image examinations such as 64-slice computed tomographic coronary angiography. Information on demography, medical history, lifestyle, and the other clinical data was collected with standardized questionnaires. This study was approved by the Ethics Committee of the First Affiliated Hospital, Guangxi Medical University (No. Lunshen 2009-Guike-018), and conducted according to the Declaration of Helsinki. Written informed consent was obtained from each subject prior to participation.

### 4.2. Biochemical Measurements

Venous blood samples of all subjects were obtained after at least 12 h of fasting, and serum was separated by centrifugation at 3000 rpm at 4 °C. Serum TC, TG (RANDOX Laboratories Ltd., Antrim, UK), HDL-C, and low-density lipoprotein cholesterol (LDL-C, Daiichi Pure Chemicals Co., Ltd., Tokyo, Japan) levels in samples were measured by enzymatic methods with commercially available kits. Serum ApoA1 and ApoB (RANDOX Laboratories Ltd.) levels were detected by the immunoturbidimetric immunoassay. All determinations were finished by an autoanalyzer (Type 7170A; Hitachi Ltd., Tokyo, Japan). The normal values in our Clinical Science Experiment Center were 3.10–5.17 mmol/L for TC, 0.56–1.70 mmol/L for TG, 0.91–1.81 mmol/L for HDL-C, 2.70–3.20 mmol/L for LDL-C, 1.00–1.78 g/L for ApoA1, 0.63–1.14 g/L for ApoB levels, and 1.00–2.50 for the ApoA1/ApoB ratio [48]. The diagnosis of type 2 diabetes mellitus was based on the World Health Organization diagnostic criteria: (1) Fasting glucose of 7.0 mmol/L or greater; (2) 2 h postprandial glucose of 11.1 mmol/L or higher; or (3) self-reported history of a physician diagnosis of diabetes or use of anti-diabetic medications [52]. Hyperlipidemia was defined as TC > 5.17 mmol/L, and/or TG > 1.70 mmol/L [48]. Hypertension was defined as a systolic blood pressure of 140 mmHg or greater, and/or a diastolic blood pressure of 90 mmHg or higher, or the use of antihypertensive drugs [53]. Smoking was defined as current smoking (yes/no). BMI was calculated as weight divided by height squared (kg/m^2^). A BMI less than 24, 24 to 28, and greater than 28 kg/m^2^ was defined as normal weight, overweight and obesity, respectively [54,55].

### 4.3. Single Nucleotide Polymorphisms (SNP) Selection and Genotyping

The selection of the SNPs was based on the following assumptions: (1) They were established by Haploview (Broad Institute of MIT and Harvard, Cambridge, MA, USA, version 4.2); (2) Information of the SNPs can be obtained from NCBI dbSNP Build 132 (http://www.Ncbi.nlm.nih.gov/SNP/); (3) The minor allele frequency (MAF) of the SNPs was higher than 1%; and (4) The SNPs might be associated with blood lipid levels and/or CHD in previous studies [56,57].

The phenol-chloroform method was used to extract total genomic deoxyribonucleic acid (DNA) from peripheral blood leukocytes. Genotypes of the rs1333040, rs1333042 and rs4977574 SNPs on chromosome 9p21, and the rs2066715 and rs2740483 SNPs in the *ABCA1* were determined by polymerase chain reaction and restriction fragment length polymorphism (PCR-RFLP) according to the previous reports [48,56,57]. The nucleotide sequences of the forward and backward primers used were 5′-CACTAGCCCAGAGAGAGGAGTGCC-3′ and 5′-TTTGGGAGCCACTGTTGT-3′ for rs1333040, 5′-TGAGTCAGCAGCAAAGGC-3′ and 5′-GC AATGCTATGGAAGGGA-3′ for rs1333042, 5′-ATGTTTGCTTTCAGGGTA-3′ and 5′-TCTGTAGTAATGGAGGTG-3′ for rs4977574, 5′-GGTAGCCCACCACTCCCCTAAAG-3′ and 5′-ATCAGCTGCCTGTCCTTGGACTA-3′ for rs2066715, and 5′-CTGCTGAGTGACTGAACTACATAAACAGAGGCCGGG**A**A-3′ and 5′-CCACTCACTCTCGTCCGCAATTAC-3′ for rs2740483 (Shanghai Sangon Biological Engineering Technology and Services Co., Ltd., Shanghai, China), respectively. The restriction enzymes of the SNPs were *Mva1269*I (rs1333040), *Sdu*I (rs1333042), *Hin6*I (rs4977574), *Taq*I (rs2066715) and *Rsa*I (rs2740483; Fermentas, CA, USA), respectively. The thermocycling protocol, the approach to electrophoresis, and the procedures for quality control were similar to a previous study [48]. In order to check the consistency and to ensure intraplate and interplate genotype quality control, a total of 10% of the samples was repeated genotyping in independent polymerase chain reactions. No genotyping discrepancies were found between the repeated samples.

### 4.4. Deoxyribonucleic Acid (DNA) Sequencing

The nucleotide sequences of the rs1333040, rs1333042, rs4977574, rs2066715 and rs2740483 SNPs were determined in Shanghai Sangon Biological Engineering Technology & Services Co., Ltd., Shanghai, China. A total of 30 samples (two samples for each genotype, respectively) detected by the PCR-RFLP were selected to submit the direct sequencing. After the PCR product was purified by low melting point gel electrophoresis and phenol extraction, the DNA sequence could be analyzed by an ABI Prism 3100 (Applied Biosyatems, Foster City, CA, USA).

### 4.5. Statistical Analyses

A statistical software package SPSS 17.0 (SPSS Inc., Chicago, IL, USA) was used to analyze the data. Mean ± standard deviation was used to express continuous variables (median and interquartile range to represent serum TG levels). Number or percentage was used to express the categorical variables. The frequency of the alleles was calculated by direct counting, and the Handy-Weinberg equilibrium was analyzed using the standard goodness-of-fit test. The differences in genotype distribution and sex ratio between the patients and controls were evaluated by *Chi*-square analyses. The clinical characteristics for the continuous variables between the patients and controls were tested by the Student’s unpaired *t*-test. The associations between the genotypes and the risk of CHD and IS, the odds ratio (OR) and 95% confidence interval (95% CI) were estimated using multivariable unconditional logistic regression. Several confounders such as age, gender, BMI, smoking, hypertension, diabetes and hyperlipidemia were adjusted for the statistical analysis. In order to illustrate the effect of the genotypes on the two diseases, five genetic models including codominant, dominant, recessive, overdominant and additive were performed for the SNPs. The interactions of the SNP-SNP on the risk of CHD and IS were detected by multifactor dimensionality reduction (MDR) 2.0 Beta 8.4 after controlling for potential confounders [58]. Bonferroni correction was employed for multiple testing (*p* < 0.01 was considered statistically significant). The LD coefficient analyses among the selected SNPs were assessed using the Haploview 4.2 program [59]. Lewomin coefficient (D’) >0.7 was considered as strong LD [60].

## 5. Conclusions

This study showed that several single nucleotide polymorphisms (SNPs) were associated with increased risk of coronary heart diseas (CHD) (rs1333042 and rs4977574) and ischemic stroke (IS) (rs4977574), and decreased risk of IS (rs2740483). The interactions of rs4977574 SNP-age (>60 year), rs2740483 SNP-BMI (>24 kg/m^2^), and SNP-SNP (rs1333040-rs1333042) on the risk of CHD and IS were observed in our study population. Genotyping for these SNPs may prove informative for the assessment of the genetic risk for CHD and IS. The results of the present study also suggest that some common variants on the chromosome 9p21 and *ABCA1* and their interactions may significantly alter the risk of CHD and IS, regardless of serum lipid levels.

## Figures and Tables

**Table 1:** Comparison of the clinical characteristics and serum lipid levels between controls and patients.

Parameter	Control	CHD	IS	*p*_1_	*p*_2_
Number	541	565	569	–	–
Male, *n* (%)	346 (64.0)	394 (69.7)	396 (69.6)	0.051	0.053
Age, years	61.39 ± 11.41	62.31 ± 10.53	62.53 ± 11.92	0.079	0.051
Body mass index, kg/m^2^	22.49 ± 2.90	23.98 ± 3.21	23.50 ± 3.60	0.000	0.000
Cigarette smoking, *n* (%)	217 (40.1)	244 (43.2)	258 (45.7)	0.300	0.078
Diabetes, *n* (%)	19 (3.5)	129 (22.8)	305 (53.98)	0.000	0.000
Hypertension, *n* (%)	123 (22.7)	282 (49.9)	326 (57.3)	0.000	0.000
Hyperlipidemia, *n* (%)	229 (42.3)	277 (49.0)	84 (14.9)	0.025	0.000
Total cholesterol, mmol/L	4.84 ± 1.07	4.55 ± 1.24	4.52 ± 1.16	0.002	0.000
Triglyceride, mmol/L	1.01 (0.69)	1.34 (0.93)	1.36 (0.94)	0.000	0.000
HDL-C, mmol/L	1.85 ± 0.51	1.14 ± 0.34	1.24 ± 0.41	0.000	0.000
LDL-C, mmol/L	2.77 ± 0.82	2.73 ± 1.04	2.67 ± 0.91	0.511	0.071
Apolipoprotein (Apo)A1, g/L	1.37 ± 0.28	1.05 ± 0.55	1.05 ± 0.55	0.000	0.000
ApoB, g/L	0.89 ± 0.21	0.90 ± 0.28	0.90 ± 0.26	0.344	0.431
ApoA1/ApoB	1.62 ± 0.55	1.40 ± 2.51	1.19 ± 0.60	0.042	0.000

CHD, coronary heart disease; IS, ischemic stroke; HDL-C, high-density lipoprotein cholesterol; LDL-C, low-density lipoprotein cholesterol. The value of triglyceride was presented as median (interquartile range), the difference between CHD/IS patients and controls was determined by the Wilcoxon-Mann-Whitney test. *p*_1_, CHD *vs*. controls; *p*_2_, IS *vs*. controls.

**Table 2:** Genotypic and allelic frequencies of the rs1333040, rs1333042, rs4977574, rs2066715 and rs2740483 SNPs in controls and patients (*n* (%)).

SNP/Group	Genotype				Allele		*p*_HWE_
	*N*	AA	AB	BB	A	B	
**rs1333040 (C>T)**							
Control	541	60 (11.1)	225 (41.6)	256 (47.3)	345 (31.9)	737 (68.1)	0.322
CHD	565	39 (6.9)	232 (41.1)	294 (52.0)	310 (27.4)	820 (72.6)	0.457
IS	569	55 (9.7)	247 (43.4)	267 (46.9)	357 (31.4)	781 (68.6)	0.846
*p*_CHD_		0.035			0.022		
*p*_IS_		0.681			0.794		
**rs1333042 (A>G)**							
Control	541	67 (12.4)	219 (40.5)	227 (42.0)	353 (32.6)	729 (67.4)	0.941
CHD	565	43 (7.60)	227 (40.2)	255 (59.2)	313 (27.7)	817 (72.3)	0.065
IS	569	56 (9.7)	243 (42.7)	270 (47.6)	355 (31.2)	783 (68.8)	0.066
*p*_CHD_		0.021			0.011		
*p*_IS_		0.376			0.470		
**rs4977574 (A>G)**							
Control	541	152 (28.1)	255 (47.1)	134 (24.8)	559 (51.7)	523 (48.3)	0.191
CHD	565	117 (20.7)	272 (48.1)	176 (31.2)	506 (44.8)	624 (55.2)	0.527
IS	569	121 (21.3)	276 (48.5)	172 (30.2)	518 (45.5)	620 (54.5)	0.599
*p*_CHD_		0.006			0.001		
*p*_IS_		0.015			0.004		
**rs2066715 (A>G)**							
Control	541	97 (17.9)	266 (49.2)	178 (32.9)	460 (42.5)	622 (57.5)	0.891
CHD	565	101 (17.9)	298 (52.7)	166 (29.4)	500 (44.2)	630 (55.8)	0.101
IS	569	116 (20.4)	279 (49.0)	174 (30.6)	627 (55.1)	511 (44.9)	0.823
*p*_CHD_		0.408			0.411		
*p*_IS_		0.510			0.256		
**rs2740483 (C>G)**							
Control	541	31 (5.70)	183 (33.8)	327 (60.4)	245 (22.6)	837 (77.4)	0.423
CHD	565	17 (2.8)	175 (31.0)	373 (66.2)	209 (18.5)	921 (81.5)	0.516
IS	569	9 (1.6)	152 (26.7)	408 (71.7)	170 (14.9)	969 (85.1)	0.223
*p*_CHD_		0.034			0.016		
*p*_IS_		0.000			0.000		

SNP, single nucleotide polymorphism; CHD, coronary heart disease; IS, ischemic stroke; HWE, Hardy-Weinberg equilibrium; AA, rs1333040CC, rs1333042AA, rs4977574AA, rs2066715AA, and rs2740483CC genotypes; AB, rs1333040CT, rs1333042AG, rs4977574AG, rs2066715AG, and rs2740483CG genotypes; BB, rs1333040TT, rs1333042GG, rs4977574GG, rs2066715GG, and rs2740483GG genotypes; A, rs1333040C, rs1333042A, rs4977574A, rs2066715A, and rs2740483C alleles; and B, rs1333040T, rs1333042G, rs4977574G, rs2066715G, and rs2740483G alleles. *p*_HWE,_ the *p* value of the Handy-Weinberg equilibrium.

**Table 3:** Genotypes of the rs1333040, rs1333042, rs4977574, rs2066715 and rs2740483 SNPs and the risk of CHD and IS in different genetic models.

SNP/Model	Ref. Genotype	Effect Genotype	CHD (OR 95% CI)	*p*	IS (OR 95% CI)	*p*
**rs1333040**						
Codominant	CC	CT	1.74 (1.06–2.86)	0.030	1.06 (0.69–1.65)	0.781
		TT	1.84 (1.13–3.01)	0.014	1.13 (0.72–1.75)	0.598
Dominant	CC	CT/TT	1.79 (1.12–2.88)	0.016	1.09 (0.72–1.66)	0.677
Recessive	CC/CT	TT	1.17 (0.90–1.52)	0.244	0.97 (0.75–1.25)	0.798
Overdominant	CC/TT	CT	1.03 (0.79–1.35)	0.820	1.07 (0.83–1.39)	0.606
Log-additive			1.23 (1.00–1.50)	0.049	1.00 (0.83–1.21)	0.994
**rs1333042**						
Codominant	AA	AG	1.73 (1.06–2.80)	0.027	1.17 (0.76–1.79)	0.483
		GG	1.92 (1.20–3.08)	0.007	1.21 (0.78–1.87)	0.395
Dominant	AA	AG/GG	1.83 (1.16–2.89)	0.010	1.19 (0.79–1.79)	0.418
Recessive	AA/AG	GG	1.24 (0.95–1.61)	0.115	1.00 (0.78–1.30)	0.974
Overdominant	AA/GG	AG	1.00 (0.76–1.30)	0.982	1.07 (0.82–1.38)	0.629
Log-additive			1.27 (1.04–1.56)	0.018	1.04 (0.86–1.26)	0.689
**rs4977574**						
Condominat	AA	AG	1.44 (1.04–2.02)	0.029	1.44 (1.05–1.98)	0.025
		GG	1.64 (1.14–2.37)	0.008	1.63 (1.14–2.33)	0.007
Dominant	AA	AG/GG	1.52 (1.11–2.07)	0.009	1.51 (1.12–2.03)	0.007
Recessive	AA/AG	GG	1.28 (0.96–1.72)	0.095	1.28 (0.96–1.70)	0.092
Overdominant	AA/GG	AG	1.11 (0.85–1.45)	0.446	1.11 (0.86–1.43)	0.427
Log-additive			1.27 (1.06–1.53)	0.010	1.27 (1.06–1.52)	0.008
**rs2066715**						
Codominant	GG	AG	1.00 (0.67–1.48)	0.994	1.10 (0.76–1.59)	0.623
		AA	1.19 (0.88–1.61)	0.260	1.00 (0.75–1.34)	0.992
Dominant	GG	AG/AA	1.14 (0.85–1.51)	0.382	1.03 (0.78–1.35)	0.849
Recessive	GG/AG	AA	0.90 (0.63–1.27)	0.537	1.00 (0.79–1.52)	0.580
Overdominant	GG/AA	AG	1.19 (0.91–1.55)	0.199	0.97 (0.75–1.25)	0.801
Log-additive			1.02 (0.85–1.24)	0.806	1.04 (0.87–1.25)	0.663
**rs2740483**						
Codominant	GG	GC	0.82 (0.61–1.08)	0.159	0.64 (0.48–0.85)	0.002
		CC	0.43 (0.22–0.83)	0.012	0.22 (0.10–0.49)	0.000
Dominant	GG	CG/CC	0.76 (0.58–0.99)	0.044	0.58 (0.44–0.76)	0.000
Recessive	GG/CG	CC	0.46 (0.24–0.89)	0.020	0.25 (0.11–0.56)	0.001
Overdominant	GG/CC	CG	0.86 (0.65–1.14)	0.305	0.69 (0.52–0.91)	0.009
Log-additive			0.74 (0.59–0.94)	0.011	0.58 (0.46–0.73)	0.000

SNP, single nucleotide polymorphism; CHD, coronary heart disease; IS, ischemic stroke.

**Table 4:** Gene-environment interactions on the risk of CHD and IS.

Factor	rs1333042 (*p*_CHD_)	rs4977574 (*p*_CHD_/*p*_IS_)	rs2740483 (*p*_CHD_/*p*_IS_)
Sex (male *vs*. female)	0.967	0.729/0.940	0.560/0.128
Age (≤60 *vs*. >60 year)	0.444	0.022/0.216	0.213/0.490
BMI (≤24 *vs*. >24 kg/m^2^)	0.565	0.720/0.824	0.198/0.035
Smoking (yes *vs*. no)	0.099	0.882/0.299	0.839/0.539
Hypertension (yes *vs*. no)	0.815	0.876/0.990	0.837/0.331
Diabetes (yes *vs*. no)	NA	0.119/NA	0.192/0.159
Hyperlipidemia (yes *vs*. no)	0.945	0.125/0.430	0.797/0.607

The *p*_CHD_ or *p*_IS_ values for interactions of genotypes and other risk factors on CHD and IS were obtained from unconditional logistic regression. BMI, body mass index; NA, not available.

**Table 5:** Gene–gene interactions on the risk of CHD.

Model	MDR Analysis				LR Analysis		
	Traing Bal.Acc	Testing Bal.Acc	CVC	*p*	Wald	OR (95% CI)	*p*
rs4977574	0.538	0.518	9/10	0.377	–	–	–
A-B	0.574	0.566	10/10	0.011	15.98	1.69 (1.31–2.19)	0.000
A-B-C	0.601	0.536	7/10	0.054	7.52	1.16 (1.04–1.73)	0.006
A-B-C-D	0.637	0.601	10/10	0.001	5.97	1.08 (1.02–1.14)	0.015

A, rs1333040; B, rs1333042; C, rs2066715; D, rs2740483; *p* value based on 1000 permutations; MDR, multifactor dimensionality reduction; CVC, Cross-validation consistency; LR analysis, Logistic regression analysis.

**Table 6:** Gene–gene interactions on the risk of IS.

Model	MDR Analysis				LR Analysis		
	Traing Bal.Acc	Testing Bal.Acc	CVC	*p*	Wald	OR (95% CI)	*p*
rs2740483	0.557	0.555	10/10	0.001	-	-	-
A-B	0.603	0.602	10/10	0.001	20.56	1.80 (1.40–2.32)	0.000
A-B-C	0.624	0.594	8/10	0.011	13.56	1.19 (0.90–1.27)	0.000
A-B-C-rs2740483	0.654	0.602	10/10	0.001	0.22	1.02 (0.94–1.40)	0.640

A, rs1333040; B, rs1333042; C, rs4977574; *p* value based on 1000 permutations; CVC, Cross-validation consistency; LR analysis, Logistic regression analysis.

## Abbreviations

ABCA1	Adenosine triphosphate-binding cassette transporter A1
Apo	Apolipoprotein
BMI	Body mass index
CHD	Coronary heart disease
DNA	Deoxyribonucleic acid
GWASes	Genome-wide association studies
HDL-C	High-density lipoprotein cholesterol
IS	Ischemic stroke
LD	Linkage disequilibrium
LDL-C	Low-density lipoprotein cholesterol
MAF	Minor allele frequency
MDR	Multifactor dimensionality reduction
MRI	Magnetic resonance imaging
PCR	Polymerase chain reaction
RFLP	Restriction fragment length polymorphism
SNPs	Single nucleotide polymorphisms
TC	Total cholesterol
TG	Triglyceride
TOAST	Trial of Org 10172 in Acute Stroke Treatment
